# Three-Dimensional FEA Analysis of the Stress Distribution on Titanium and Graphene Frameworks Supported by 3 or 6-Implant Models

**DOI:** 10.3390/biomimetics8010015

**Published:** 2023-01-01

**Authors:** Shrikar R. Desai, Kiran Deepak Koulgikar, Nasser Raqe Alqhtani, Ali Robaian Alqahtani, Abdullah Saad Alqahtani, Adel Alenazi, Artak Heboyan, Gustavo V. O. Fernandes, Mohammed Mustafa

**Affiliations:** 1 Department of Periodontology and Implantology, HKE’S S. Nijalingappa Institute of Dental Sciences and Research, Kalaburagi 585105, India; 2 Department of Oral and Maxillofacial Surgery and Diagnostic Sciences, College of Dentistry, Prince Sattam Bin Abdulaziz University, Al-Kharj 11942, Saudi Arabia; 3 Department of Conservative Dental Sciences, College of Dentistry, Prince Sattam Bin Abdulaziz University, Al-Kharj 11942, Saudi Arabia; 4 Department of Preventive Dental Sciences, College of Dentistry, Prince Sattam Bin Abdulaziz University, Al-Kharj 11942, Saudi Arabia; 5 Department of Prosthodontics, Faculty of Stomatology, Yerevan State Medical University after Mkhitar Heratsi, Str. Koryun 2, Yerevan 0025, Armenia; 6 Periodontics and Oral Medicine Department, University of Michigan School of Dentistry, 1011 North University Ave, Ann Arbor, MI 48109, USA

**Keywords:** endosseous implants, finite element analysis, graphene, mandible, titanium

## Abstract

Titanium is the main component of dental implants. It is also routinely used as a framework material for implant-supported full-arch prostheses due to its low density, biocompatibility, and other mechanical properties. Remarkable mechanical properties such as lesser mass density and higher young’s modulus of graphene have gained popularity among scientists, improving the properties of biomedical implants. Thus, our study aimed to compare the outcome through the von Mises stresses generated on All-on-6 and All-on-3 implant models, as well as on the framework, and evaluate the effect of stress patterns on the crestal bone around implants in the mandible. FEA (Finite Element Analysis) study was carried out using edentulous mandible models. Four 3D FEA models with 3 and 6 implants were used (Model 1: Titanium bar-supported 6 straight implants; Model 2: Graphene bar-supported 6 straight implants; Model 3: Titanium bar-supported 3 implants with 30 degrees-tilted; Model 4: Graphene bar-supported 3 implants with 30 degrees-tilted) in order to simulate endosseous implant designs. The implant measuring 4.2 mm in diameter and 11.5 mm in length were used. The most distal implants in the 3-implant models were placed with angulation of 30 degrees; in 6 implants, they were vertically placed. All the models were analyzed for vertical and oblique axis with a single force magnitude of 100 N. In all four implant models and under loading conditions, the peak stress points were always on the neck of the most distal implant. von Mises stresses were within the normal stress range. In a conventional six-straight implant model supported by a titanium framework, the cortical stress in the region of implants was 25.27 MPa, whereas, in the graphene framework, it was 12.18 MPa. Under vertical load, there was a significant difference in the cortical stress around the tilted implants (30 degrees) in the 3-implant system of titanium and graphene frameworks, respectively, 70.31 MPa and 21.27 MPa. The graphene framework demonstrated better results than the titanium framework for the conventional six-implant system under vertical load, achieving stress of 30.09 MPa and 76.60 MPa, respectively. In the case of the 3-implant system, a significant difference in the bar stress was observed between graphene and titanium, respectively, 256.32 MPa and 180.1 MPa of bar stress. Within the limitation of this study, the peri-implant stresses were decreased using graphene framework models. Hence, it was possible to conclude that the best load-bearing capacity results were found in the graphene framework group compared to the titanium framework for All-on-6 and All-on-3 implant models, even though both materials are reliable options used as framework materials in implant-supported full-arch prostheses.

## 1. Introduction

Bone loss has a substantial harmful impact on the oral health of patients. This also perpetuates the increase in the patient’s need for rehabilitation with full-mouth fixed prostheses. Studies have shown that treatment with implant-supported full arch prosthesis significantly alleviates edentulous patient problems [1]. Eliasson et al. in their study demonstrated that the insertion of four implants in the anterior mandible was advocated as a treatment alternative to the placement of 6 or 8 implants. However, when the implants were placed in the anterior mandible, this design increased the cantilever length and stress over the distal-most implant [2]. To rectify this problem, some authors put forward the concept of tilted implants where the distal-most implants were tilted which led to the broadening of the prosthetic load-bearing area, increase in the primary stability, and making it easy to use the lengthier implants [3].

The patients often refused prosthetic fabrication as more implants were used, resulting in a more complex and costly rehabilitation phase. Hence, there is a need to reduce the number of implants, which will not only cut down the cost but also shortens the surgical treatment time and minimizes trauma [4]. Although the quantity of implants affects the peri-implant stress distribution, it has been shown that fewer implants can also slightly increase the stress in the abutment and screws [5]. Nevertheless, prosthetic frameworks and abutments may compensate for the above biomechanical weaknesses [6]. An appropriate prosthetic framework is required in edentulous patients. Studies have shown that fracture, bending, and screw loosening problems are associated with either poor or ill-fitting framework material. The type, position, and material used as a prosthetic framework influence the loading stress pattern as it transfers the stress to the underlying dental implants and the bone and, in the long term, can consequently lead to the resorption of bone around the implants, ultimately leading to its failure. So, selecting a prosthetic framework that can dampen these excessive loads and transfer the favorable loads onto the underlying bone becomes obligatory [7,8,9,10].

Titanium has always been one of the leading materials of choice for the prosthetic framework as it has favorable biomechanical properties such as biocompatibility and low density and is economical [11]. Using a 2D in silico model, the contact pressure for metal-on-metal bearings of an all-metal hip implant was successfully evaluated by Jamari, J. et al. Ti6Al4V-on-Ti6Al4V bearings demonstrated the most significant reduction in contact pressure due to their reduced wear. This material was chosen due to its superior biocompatibility and corrosion resistance compared to CoCrMo, SS 316L [12]. Based on numerical simulations, Heboyan et al. investigated different stiffnesses of the superstructure for the support of zygomatic implants. Material stiffer than polymeric superstructures, such as zirconia, CoCr, and titanium, demonstrated improved mechanical performance [13]. Recently, Graphene material has gained importance under its extraordinary mechanical properties. It is used as a coating material for titanium alloy in orthopedic applications. It showed promising results in terms of stiffness, low density, higher young’s modulus, biocompatibility, and enhanced bone-implant bonding strength [14]. Moreover, it is a material made up of asymmetrical carbon atoms, has antibacterial properties against Streptococcus aureus, and increases bone formation and fastidious maturing of the bone in animal studies [15].

FEA is the most reliable and easy method to determine the applied force at any given time. It is also a mathematical tool to predict stresses in the peri-implant area and around the components of implant-supported constructions. It was first implemented in the mid-1960 to solve aeronautic engineering problems, pioneered by Weinstein, who applied this method in dental implants and their biomechanics [16]. The FEA determines the abnormal stresses responsible for implant failure, named von Mises stresses. These are the combination of 3 principal stresses at a given point [17,18]. Since 1999, when P-I Branemark and colleagues introduced the Branemark Novum concept, three implant systems have been used to restore edentulous mandibles with full-arch prostheses [19]. As a result of advancements in implant fabrication technology, Branemark Novum implants are not currently used in dental practices. There is still uncertainty regarding the safety of using three implants to support full-arch implant-fixed prostheses for a prolonged period (the “all-on-three” concept). Based on Brando TB et al. systematic review, the survival rate for implants and prosthetics was 95.43 percent and 89.66 percent, respectively. Most prosthetic failures were associated with mobile implants, suggesting that when the “all-on-three” concept is applied, the prosthesis’ longevity is directly related to the implant’s ability to support the prosthesis [20]. In a research study conducted with an in vivo model, De Bryun H. and colleagues concluded that there is not much difference in axial force between three and four supporting implants. With fewer supporting implants, however, the bending moments became significantly higher, resulting in the failure of the all-on-three implants. In none of the studies graphene was used as a framework material [21]. Hence, the objective of our study was to compare the outcome of von Mises stresses generated on all-on-6 and all-on-3 implant models as well as on the framework and to study the effect of stress patterns on mandibular crestal bone around implants.

## 2. Materials and Methods

Based on ISO 10303-107:2019, 3D finite element models (FEM) comprised edentulous mandibles. There were 6 conventional implants vertically positioned and a 3-implant system placed within the interforamina region with 30 degrees angulation. Each model consists of a single implant setting with various implants. Computerized tomography (CT) of the lower jaw was performed to obtain the FEM of the mandible.

**Figure 1:**
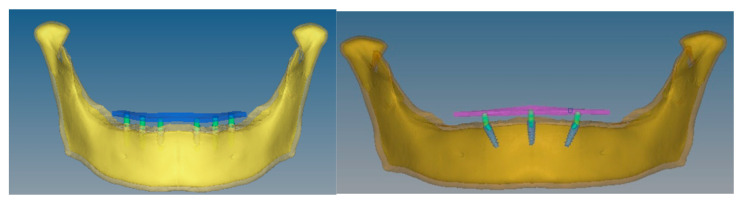
Virtual geometric models (VGM) for 6 (**Left**) and 3-implant (**Right**) systems.

The first procedure was to create the Virtual Geometric Models (VGM) (Figure 1). The VGM has a close resemblance with the actual structures, and it produces more reliable results. Three-dimensional models were incorporated as it allows the complete assessment of structure and loads in any direction. Ansys R v.18.1 software was used to visualize and segment CT images. The 3D tetrahedral structural solid finite elements (Solid Edge software, v.19) were used for modeling the bone, abutment, framework, implant, and occlusal surface of the biomaterial. Computer Intel Core i7 with 16 GB RAM and Windows 10 system was used to carry out the study. It took around 10 min for the execution of each model. The solver section is a crucial component of FEA, which uses numerical methods to solve millions of equations taken from the pre-processor. The solver adopted in this study was the implicit solver. There was homogeneity, isotropic, and linear elasticity between the materials used in the models.

### 2.1. Bone Characteristics

The bone’s quality was considered density 1 (D1), with a minimal amount of cancellous bone [22]. It was modeled as follows: (i) height—17 mm; (ii) width—minimum of 8 mm; and (iii) interforamina distance—46 mm. The cortical bone’s top and bottom layers of 2 mm and 3 mm, respectively, with an intervening layer of 12 mm thickness of trabecular bone, were modeled [23]. Properties of cortical and cancellous bone were assigned in Table 1 [24].

### 2.2. Implants Setting

Solid implants of length (11.5 mm) and diameter (4.2 mm) were used in this study. The 30-degree-tilted implants of the 3-implant system were placed in the interforamina region. The bone-implant contact was considered as having complete osseointegration as a delayed loading system.

### 2.3. Groups of Study

The following four 3D working models were constructed for the analysis. **Model 1**: Titanium bar-supported 6 straight implants; **Model 2**: Graphene bar-supported 6 straight implants; **Model 3**: Titanium bar-supported 3 implants with 30 degrees-tilted; **Model 4**: Graphene bar-supported 3 implants with 30 degrees-tilted.

### 2.4. Loading

The present study was a linear elastic static study. Here, the load is assumed to be static and material properties are isotropic. All four 3D models were subjected to a 100 N magnitude force. Both vertical and oblique forces were applied. The bilateral vertical forces depicted a clenching situation, and the unilateral showed the masticatory forces. The oblique force was applied unilaterally at an angle of 30 degrees to the long axis [3].

### 2.5. Meshed Models

**Figure 2:**
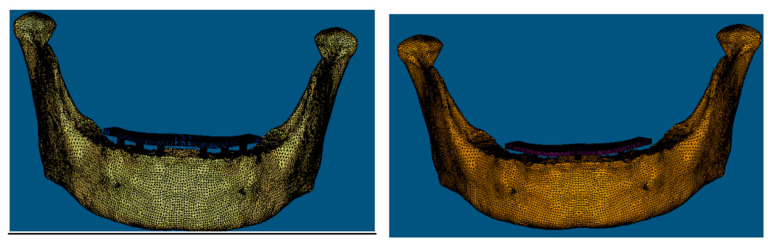
Meshed models for 6 (**Left**) and 3 (**Right**) implant systems.

The 3D models were meshed using meshing software (Hypermesh, v.13.0) (Figure 2). Meshed models are also known as finite element models, consisting of nodes and element data. The accuracy of the finite element solution is directly proportional to the number of nodes and elements. The assembly meshed with 10-node tetrahedral elements. Since the structure is complex, it was not possible to apply hexamesh; then, the tetrahedral mesh was used with an element of 0.5 mm. In total, 670,451 elements and 950,699 nodes were incorporated into the mesh and refined in the possible stress concentration regions. Essential factors, such as Poisson’s ratio, Young’s modulus, and the densities of each material, were included in the mesh. Here, intimate contact was considered between the implant and bone.

### 2.6. Framework

**Figure 3:**
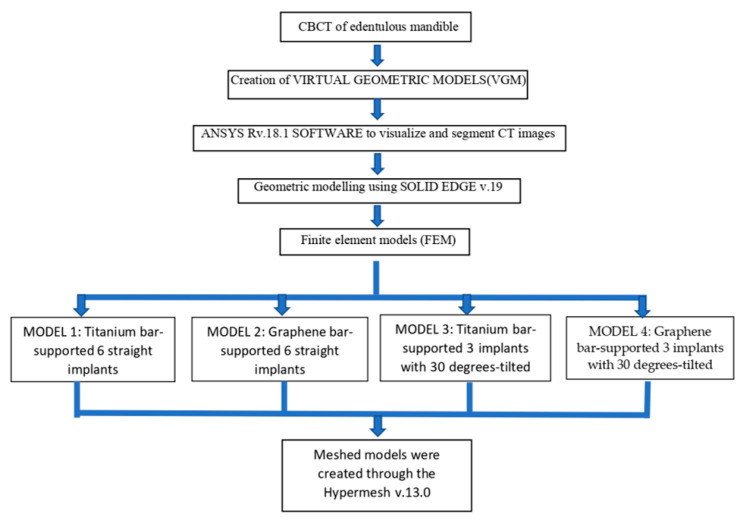
Flowchart depicting the study workflow.

Titanium has always been the material of choice for prosthetic framework material. Graphene, with its multiple forms, has shown biocompatibility for many applications as in tissue engineering, drug delivery, implants, and biosensing [24]. Graphene’s rigidity and roughness are attributed to its asymmetric nanostructure [25]. It promotes adhesion, proliferation, and osteogenic differentiation, thereby improving the biological and mechanical properties of the implants, as validated in various studies [26,27]. Table 1 shows the mechanical properties of titanium and graphene, which are used as framework materials in the present study [14]. Flowchart depicting the study workflow is given in Figure 3.

## 3. Results

The data obtained from the von Mises stress for All-on-6 and All-on-3 implant models and frameworks permitted their assessment and comparison. In our study, the maximum stress points were always found at the neck of the most distal implant in all the models of load simulations. The von Mises stresses were within the range of normal stresses. The following stresses were evaluated: (i) Cancellous stress in the region of implants; (ii) Cortical stress in the region of implants; (iii) Stress on the implants; and (iv) Bar stress.

### 3.1. Cancellous Stress in the Region of Implants (Graph 1)

**Figure 4:**
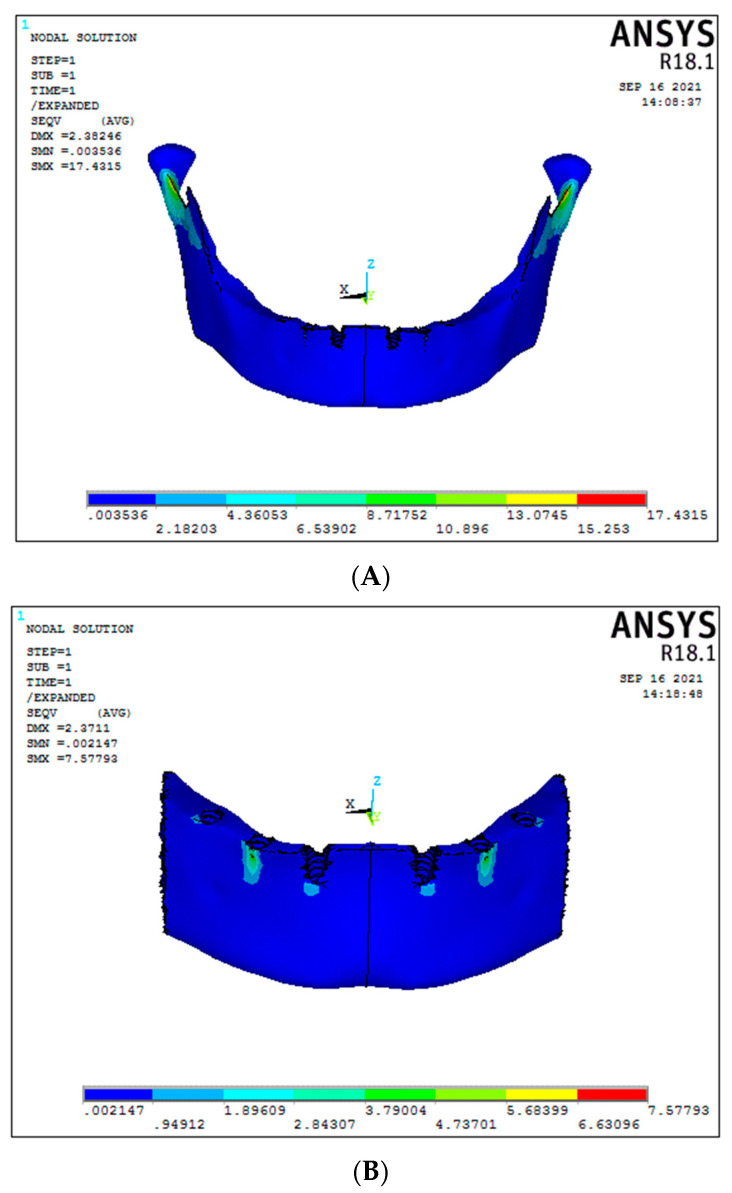

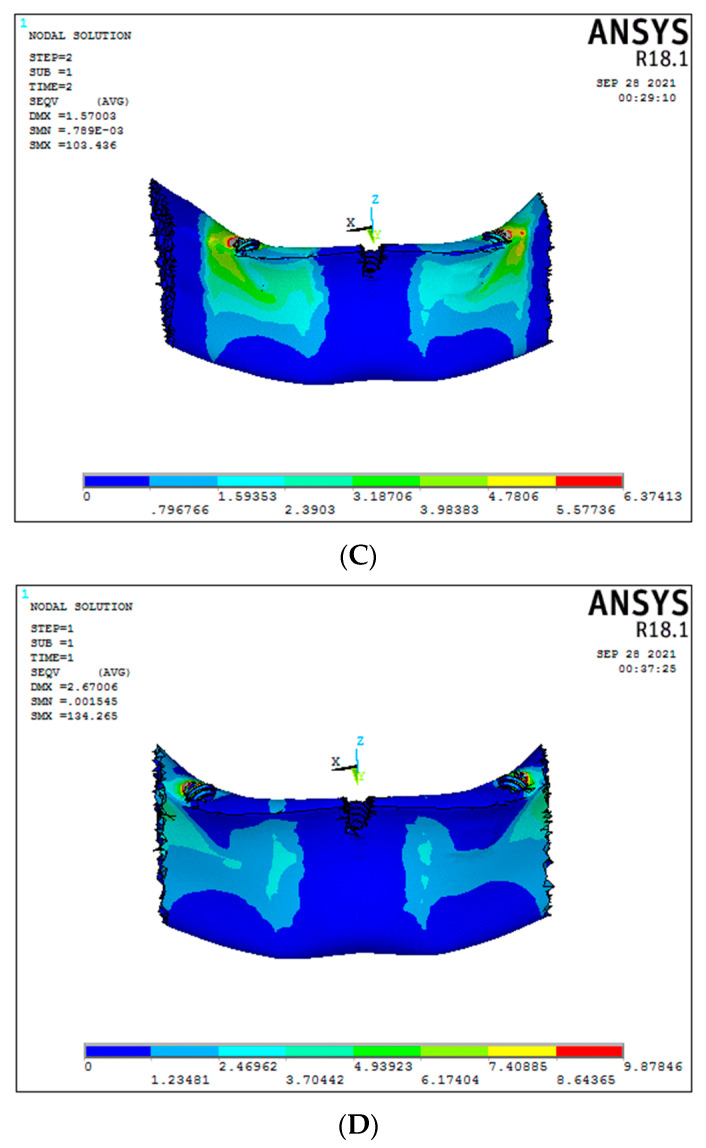
Cancellous stresses under vertical load ((**A**) Model 1; (**B**) Model 2; (**C**) Model 3; (**D**) Model 4).

**Figure 5:**
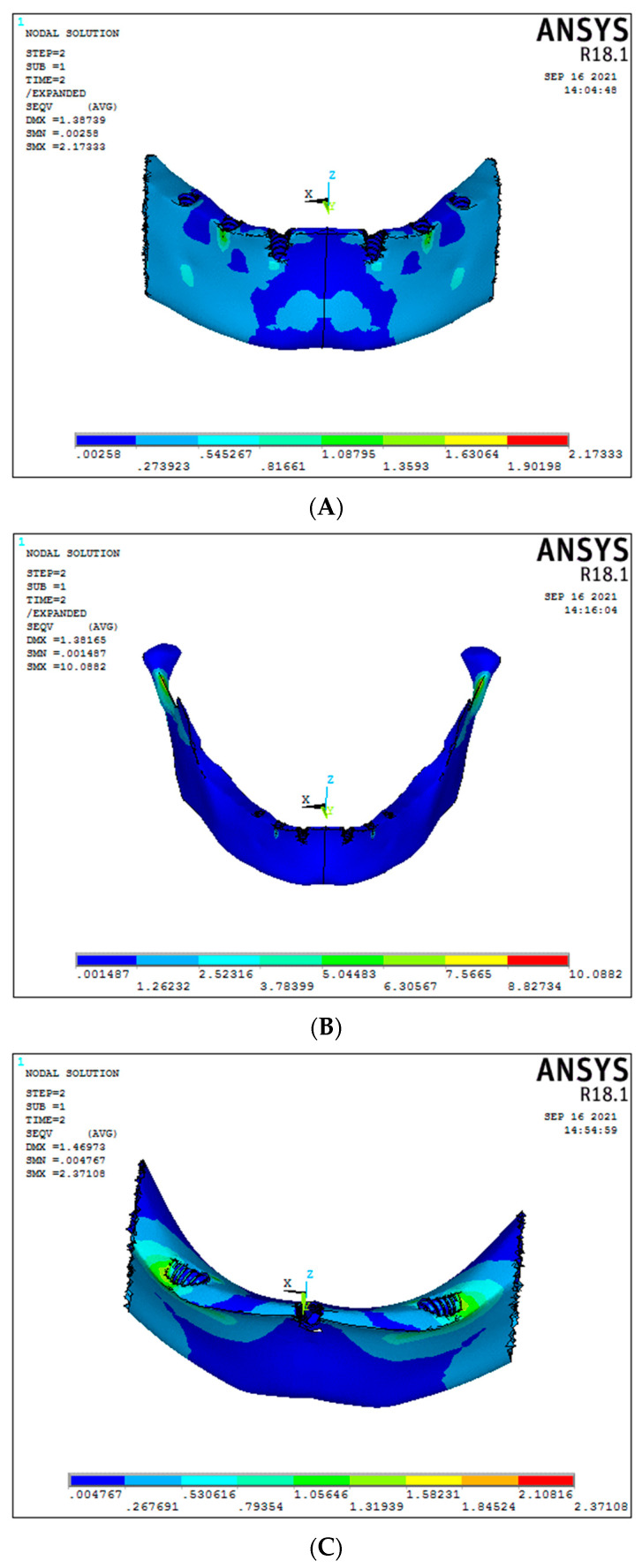

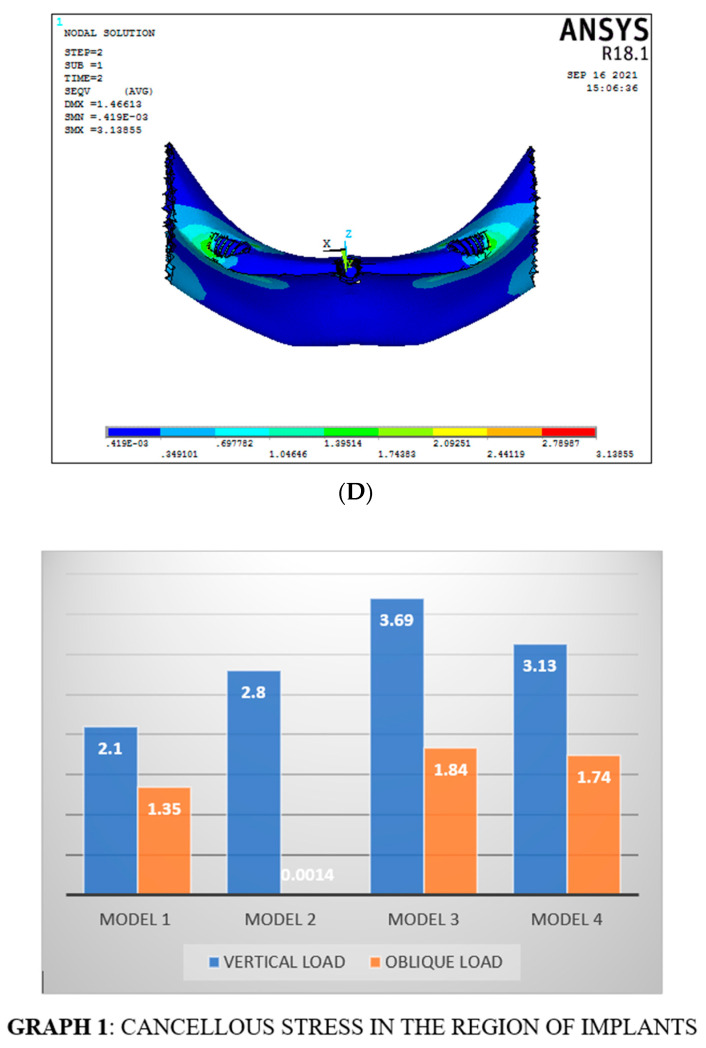
Cancellous stresses under oblique load ((**A**) Model 1; (**B**) Model 2; (**C**) Model 3; (**D**) Model 4).

Under vertical load, the cancellous stress was 2.1 MPa for Model 1, i.e., the conventional 6 straight implant model of titanium framework (Figure 4A); whereas for Model 2, graphene framework, it was found 2.8 MPa (Figure 4B). No statistically significant differences were seen in both models under vertical loading. In the 30-degree tilted of the 3-implant system, cancellous stress under vertical load was 3.69 MPa and 3.13 MPa, respectively, for titanium and graphene frameworks (Figure 4 C,D). No significant difference was found in both models (Figure 5) (Graph 1).

Under oblique forces, the stress in the cancellous region of implants was 1.35 MPa and 0.0014 MPa for conventional 6 straight implant models, respectively, for the titanium and graphene frameworks, with graphene showing better stress distribution compared to titanium framework models (Figure 5A,B). Evaluating the 30-degree tilted implants in the 3-implant system, the cancellous stress under oblique load was 1.84 MPa and 1.74 MPa in titanium and graphene frameworks, respectively (Figure 5C,D). No significant difference was seen in both models.

### 3.2. Cortical Stress in the Region of Implants (Graph 2)

**Figure 6:**
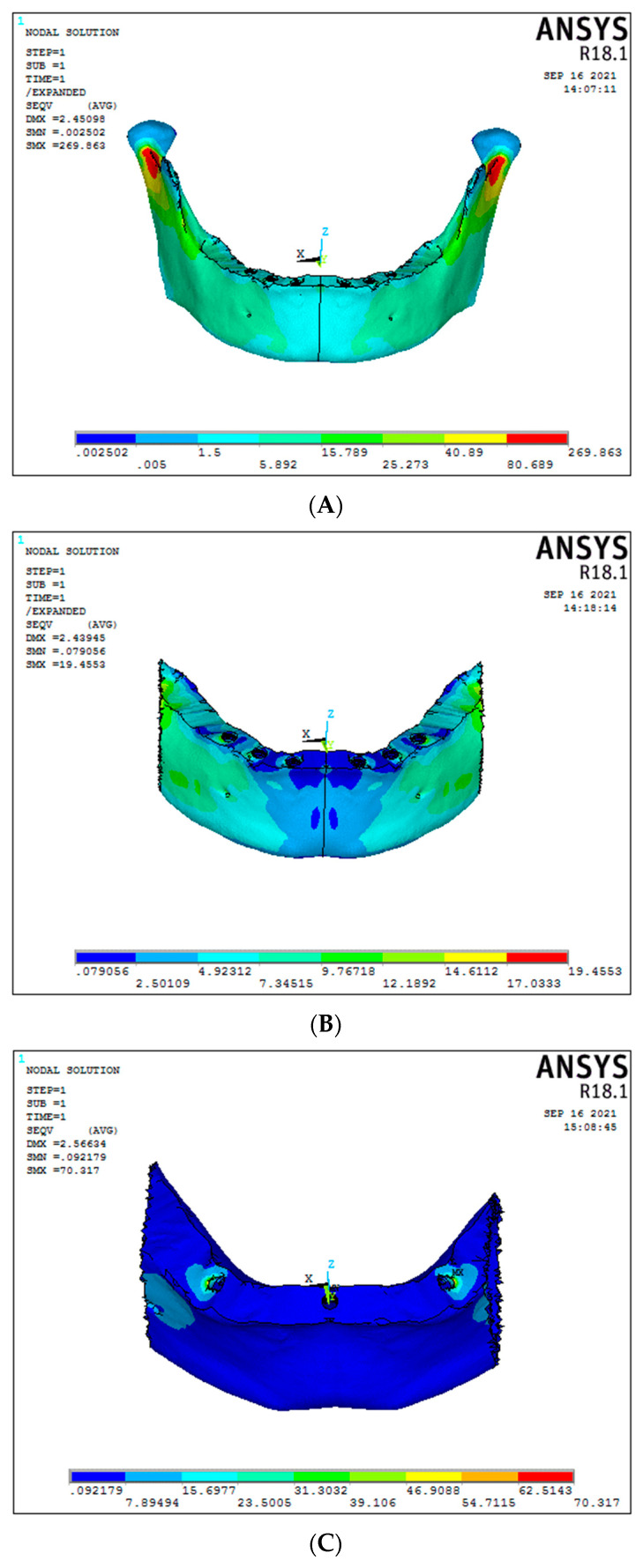

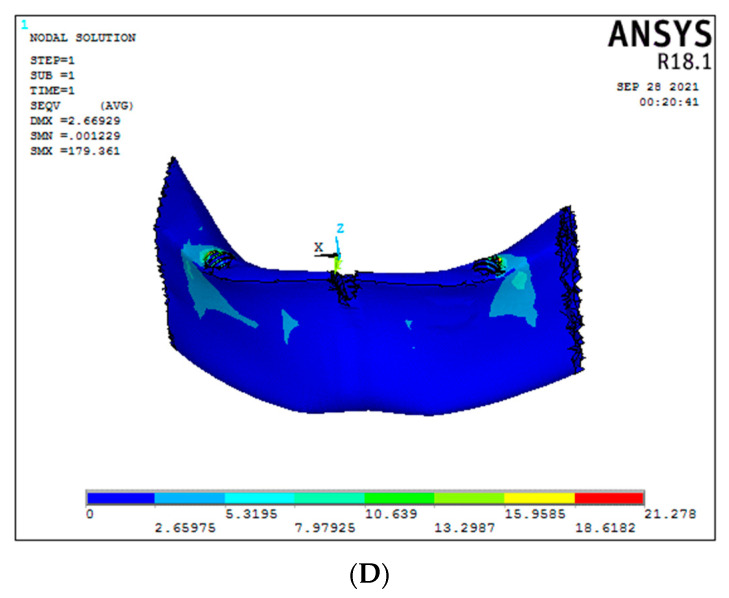
Cortical stresses under vertical load ((**A**) Model 1; (**B**) Model 2; (**C**) Model 3; (**D**) Model 4).

Under vertical load, the cortical stress in the region of implants of Model 1 (conventional 6 straight implant model supported by a titanium framework) was 25.27 MPa (Figure 6A). In contrast, the graphene framework (Model 2) had 12.18 MPa (Figure 6B). A significant decrease in cortical stress was seen in the graphene framework compared to the titanium framework. A significant difference was seen in the 30-degree tilted implants for the 3-implant system, presenting cortical stress around the implants for titanium and graphene frameworks, respectively, of 70.31 MPa and 21.27 MPa (Figure 6C,D), (Graph 2).

**Figure 7:**
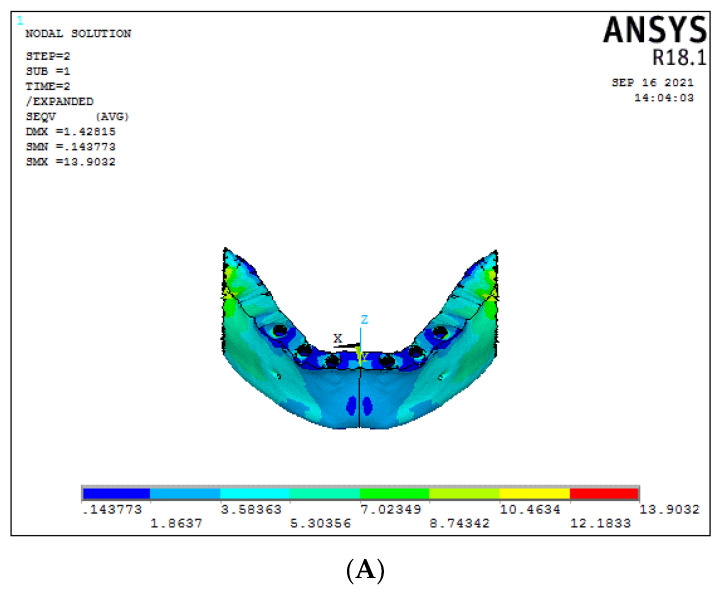

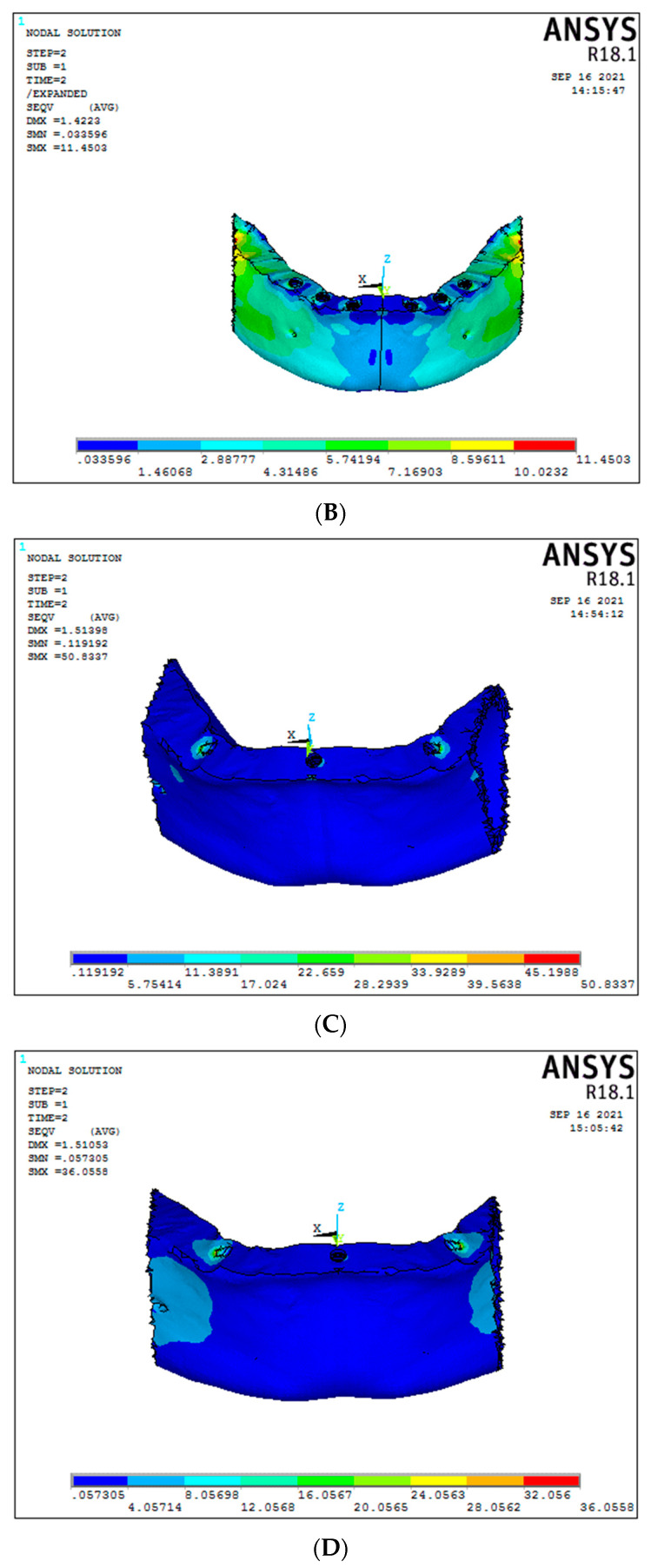

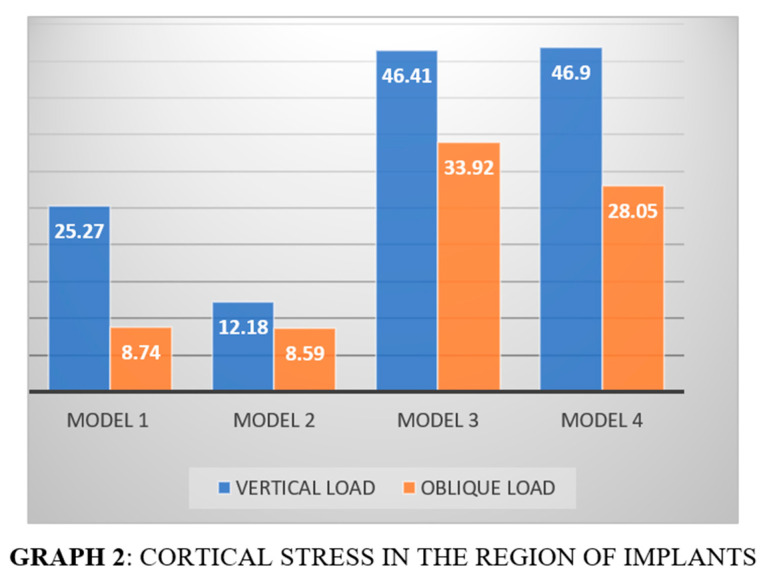
Cortical stresses under oblique load ((**A**) Model 1; (**B**) Model 2; (**C**) Model 3; (**D**) Model 4).

The cortical stress for Model 1 and Model 2 (respectively, conventional six-straight implant system supported by titanium and graphene framework) under oblique load was 8.74 MPa and 8.59 MPa. No significant difference was noticed in cortical stress (Figure 7A,B). Otherwise, in the case of 30-degree tilted implant models, a significant difference in the cortical stress was seen between titanium and graphene models, showing, respectively 33.92 MPa and 28.05 MPa, with graphene presenting a better cortical stress distribution (Figure 7C,D).

### 3.3. Stress around the Implants (Graph 3)

**Figure 8:**
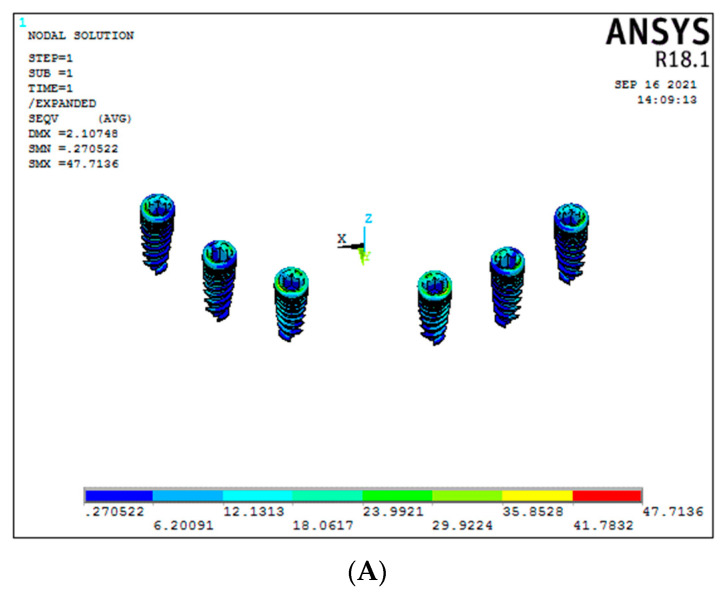

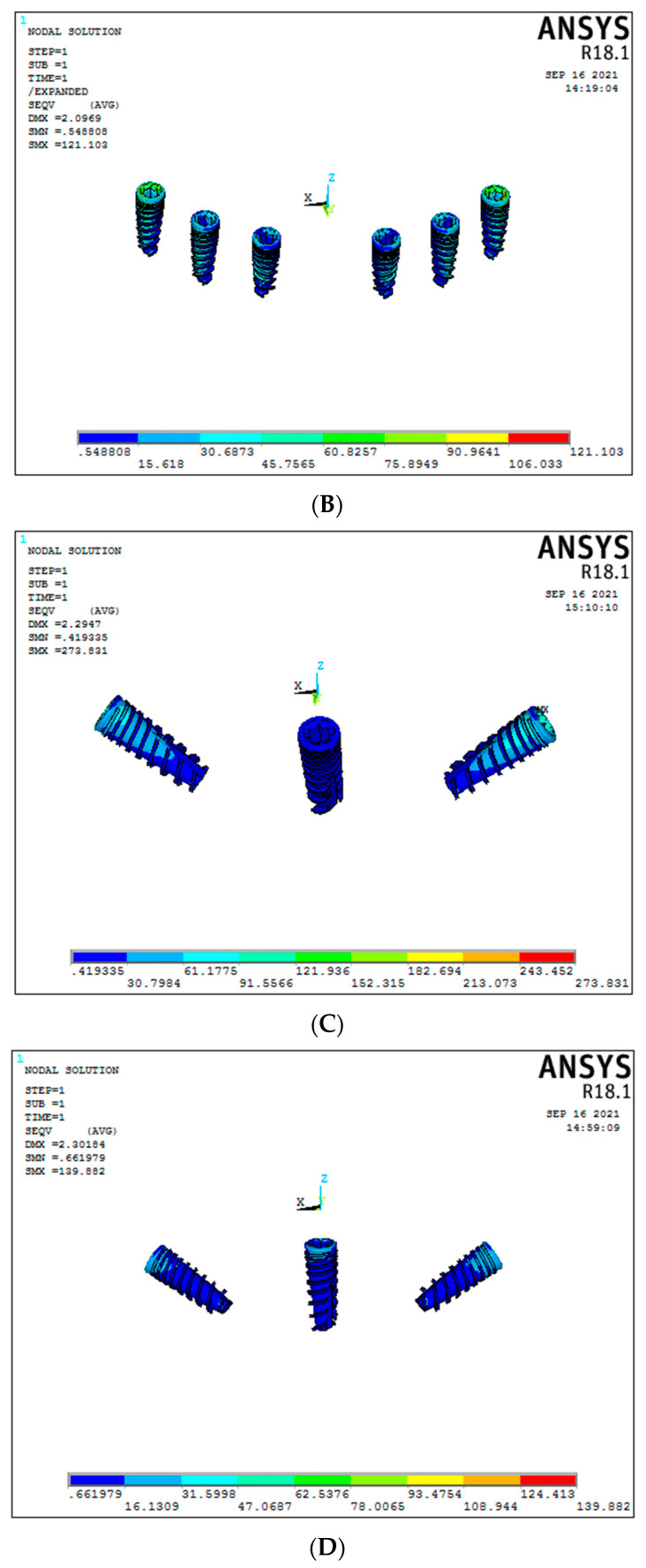
Stresses on the implants under vertical load ((**A**) Model 1; (**B**) Model 2; (**C**) Model 3; (**D**) Model 4).

Under vertical load, a significant difference was seen between titanium and graphene framework models. In the conventional six-implant system with graphene showed significant results of 35.85 MPa (Model 2) compared to the titanium framework (Model 1), which showed 75.89 MPa of stress around the implants (Figure 8A,B). Similar results were seen even for the 3-implant systems, with graphene (Model 4) showing significant results compared to titanium framework (Model 3), respectively, 31.59 MPa and 243.45 MPa (Figure 8C,D), (Graph 3).

**Figure 9:**
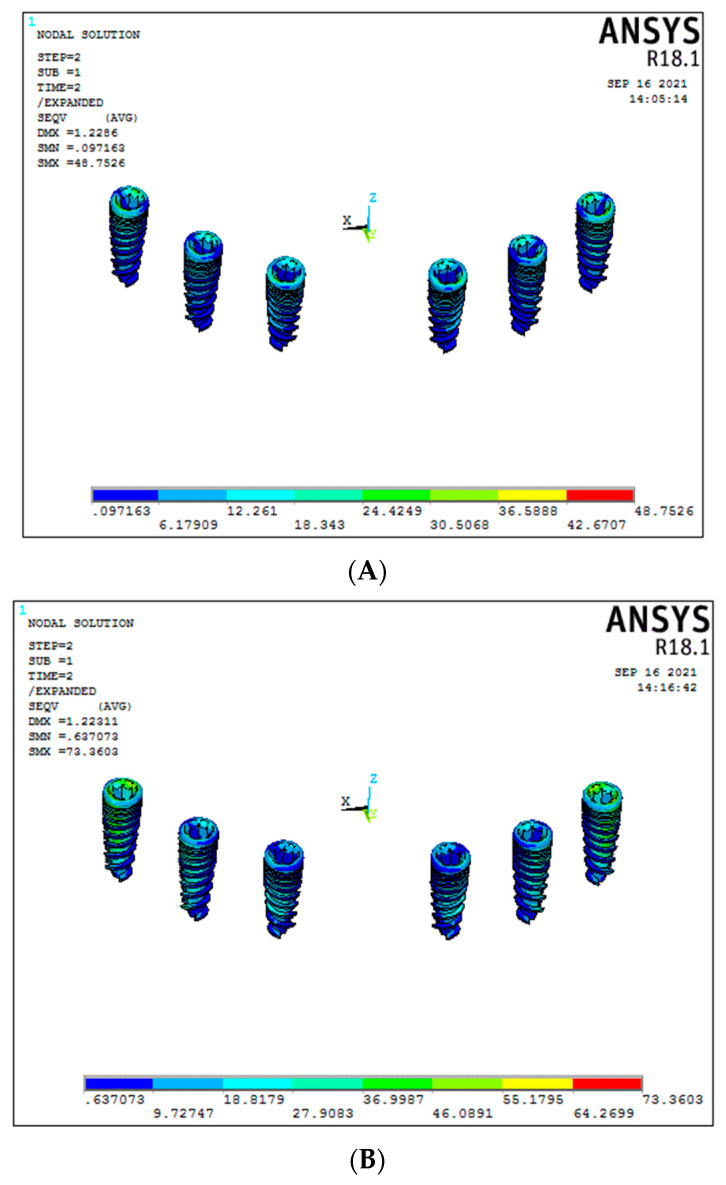

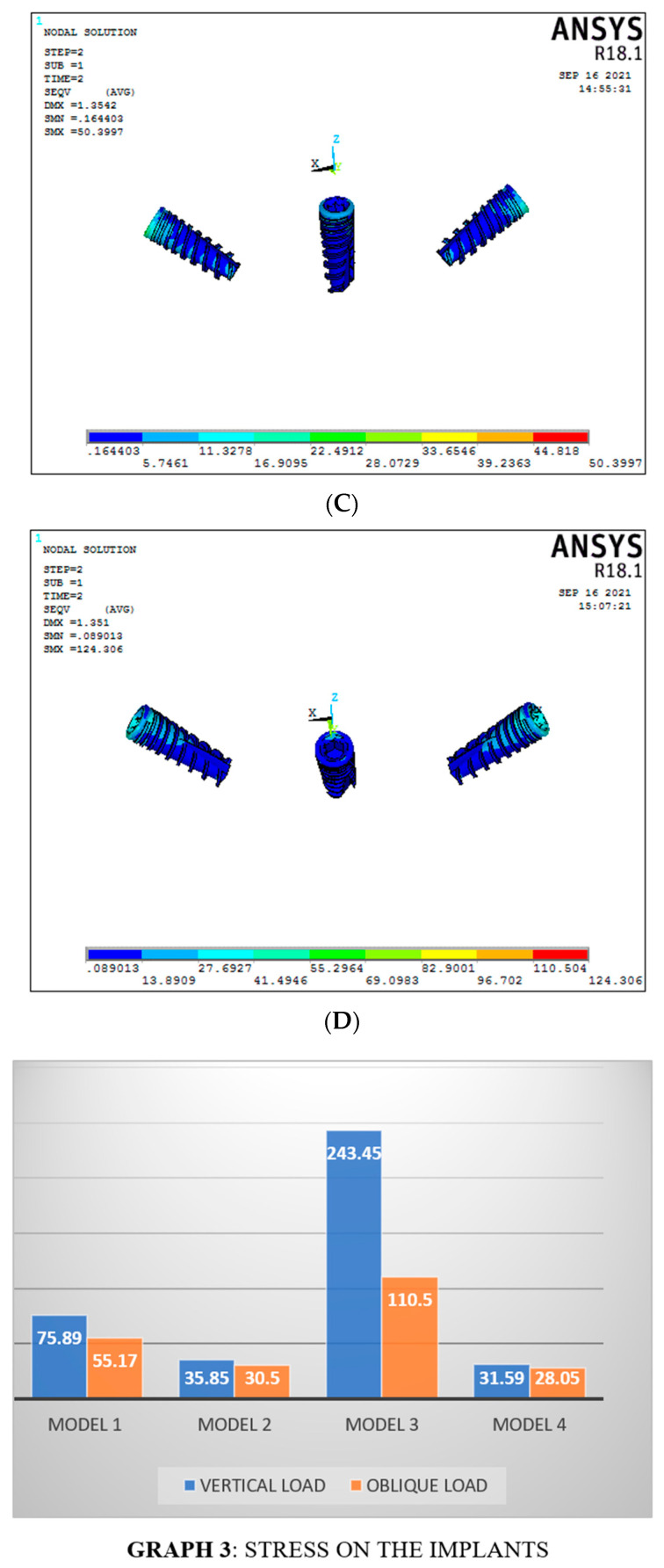
Stresses on the implants under oblique load ((**A**) Model 1; (**B**) Model 2; (**C**) Model 3; (**D**) Model 4).

For the oblique load applied, graphene showed better results than titanium (conventional six-implant systems), respectively, 30.5 MPa and 55.17 MPa (Figure 9A,B). In contrast, in Models 3 and 4 (3-implant systems), with the same oblique load, there was a significant difference between them, with graphene showing significant results (33.65 MPa) compared to the titanium group (110.50 MPa) (Figure 9C,D).

### 3.4. Bar/Framework Stress (Graph 4)

**Figure 10:**
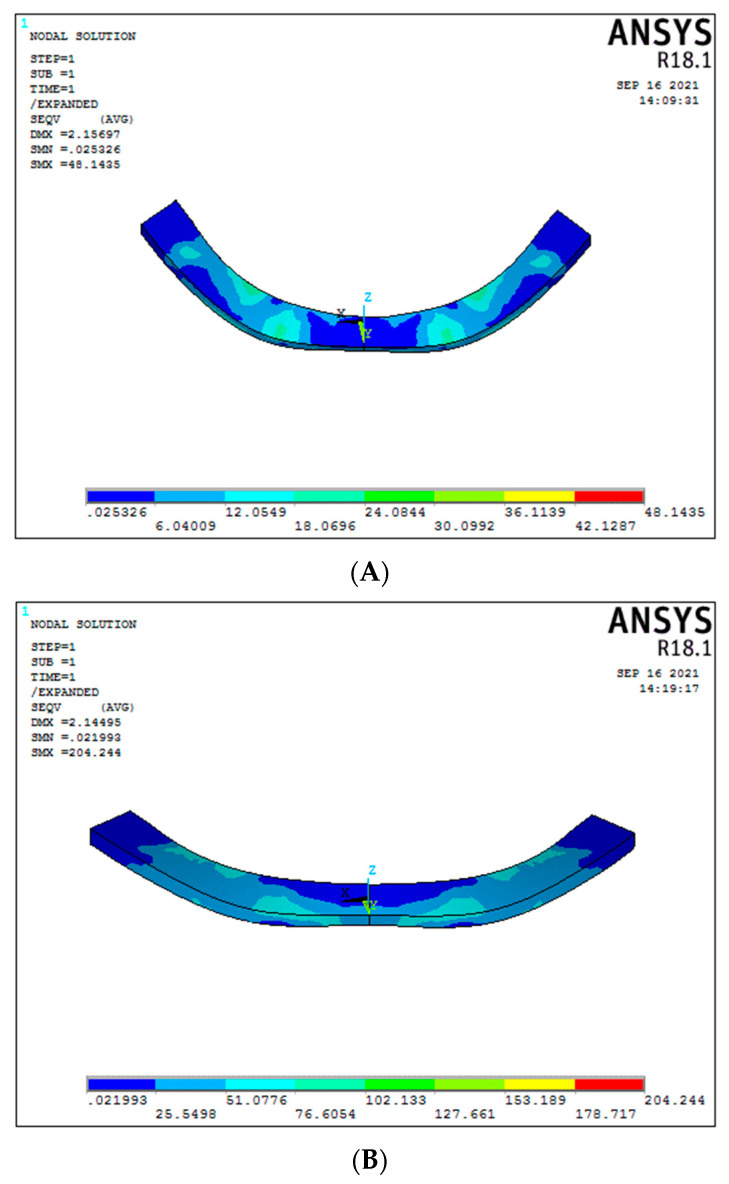

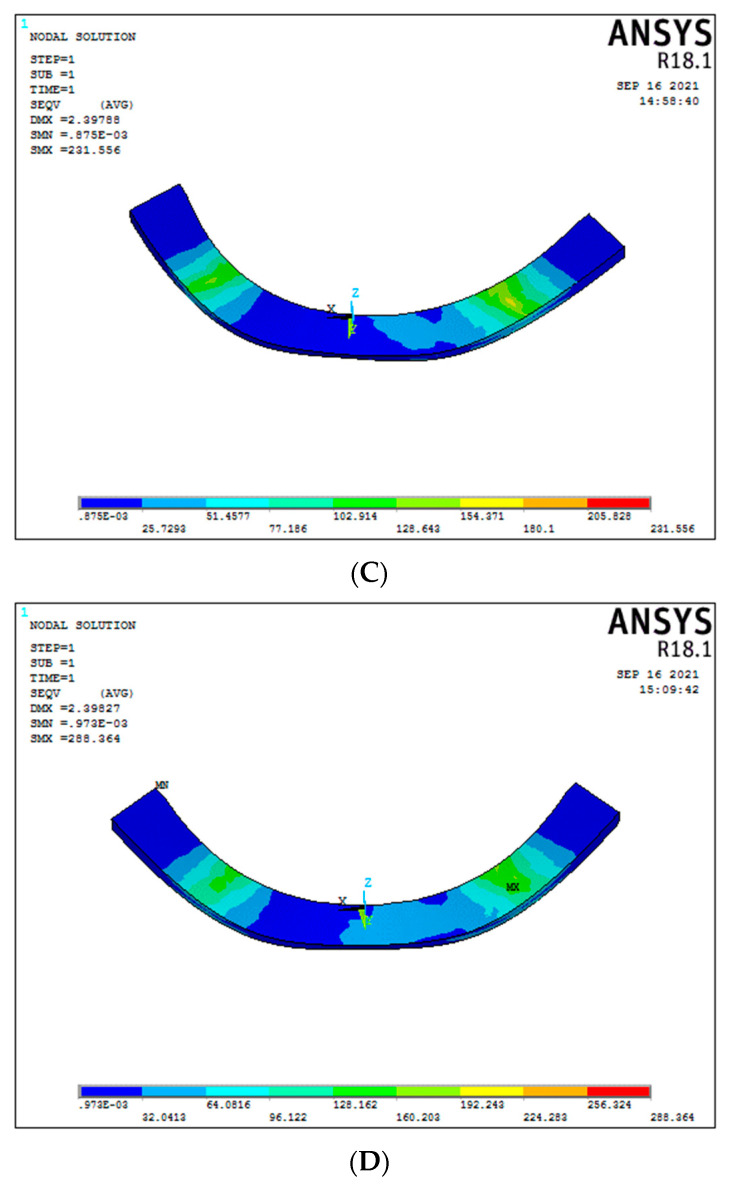
Bar stress under vertical load ((**A**) Model 1; (**B**) Model 2; (**C**) Model 3; (**D**) Model 4).

In the conventional 6-implant systems under vertical load, graphene (Model 2) had better and higher stress results than titanium framework (Model 1), respectively, 76.60 MPa and 30.09 MPa (Figure 10A,B). For the 3-implant systems, a statistically significant difference in the bar stress was found between graphene (Model 4) and titanium (Model 3), respectively, 256.32 MPa and 180.1 MPa (Figure 10C,D), (Graph 4).

**Figure 11:**
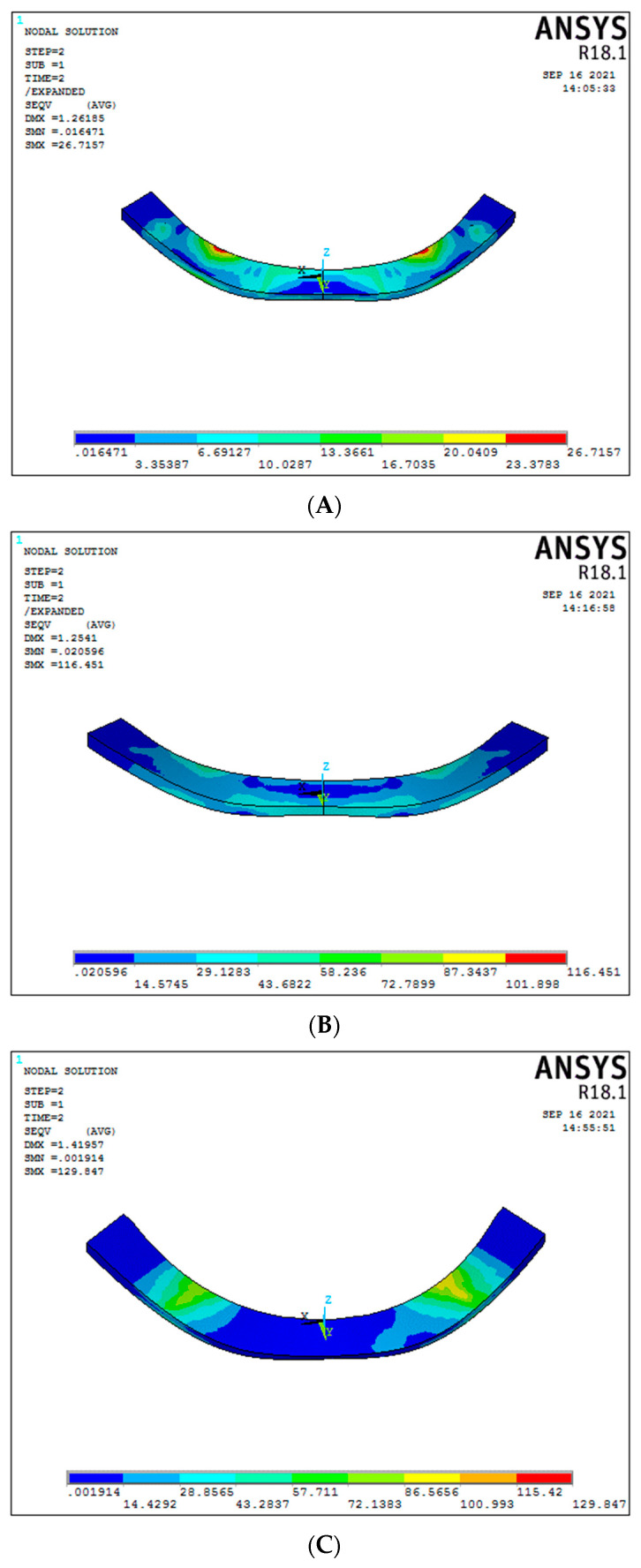

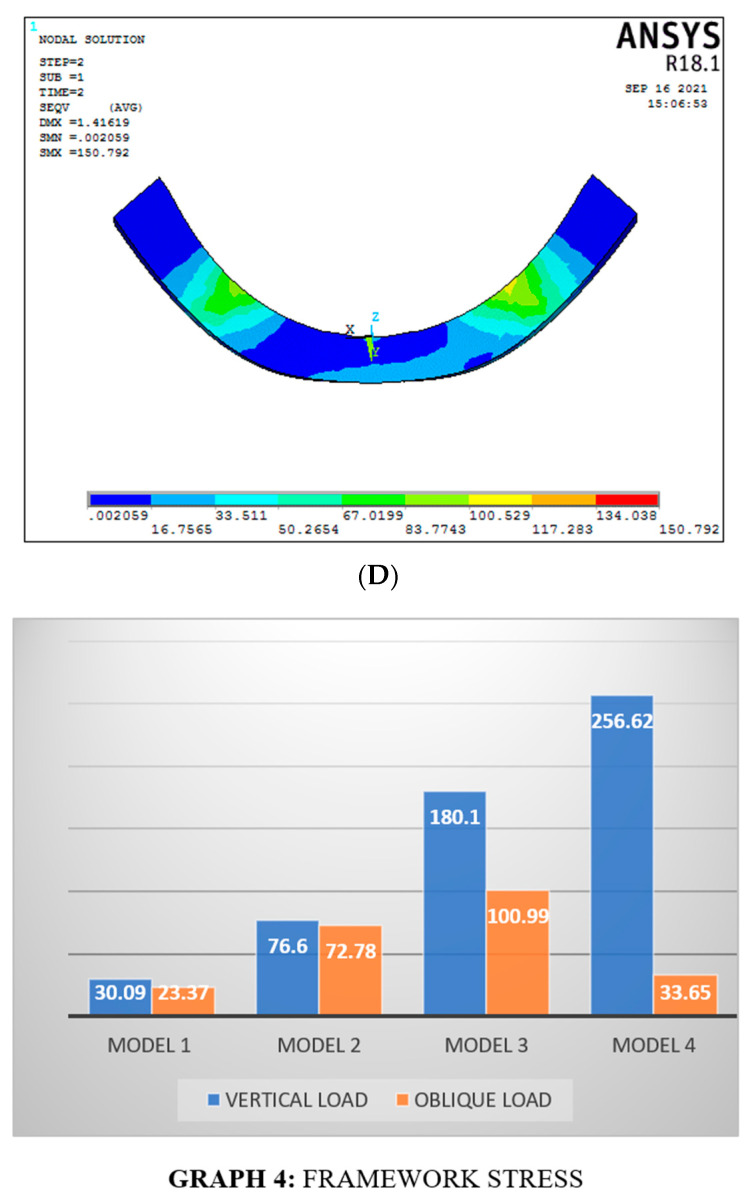
Bar stress under oblique load ((**A**) Model 1; (**B**) Model 2; (**C**) Model 3; (**D**) Model 4).

Considering the oblique load, the framework stress in a conventional 6-implant system was 23.37 MPa for titanium and 76.60 MPa for the graphene framework (Figure 11A,B). On the other hand, in the 3-implant systems, the bar stress for oblique forces was 100.99 MPa and 33.65 MPa for titanium and graphene, respectively (Figure 11C,D). The graphene framework under oblique load had significant results, suggesting better load-bearing capacity than titanium.

## 4. Discussion

All-on-4 concept advocated by Malo et al. involved a framework that retained implant-supported full arch prosthesis by placing 4 implants in the anterior maxilla and mandible. This Malo’s concept uses 45 degrees of angulation for the tilted implants. In their study, it was concluded there was no increase in the local stress due to the angulation of the implants which are splinted. An increase in angulation did not increase the von Mises stresses [28].

The more implants, the more difficult and expensive the oral rehabilitation. Brånemark and colleagues, in 1999, presented the Brånemark novum concept, which implemented three implants to restore the edentulous mandible with full arch prosthesis. The protocol of this concept consisted of a two-stage surgical procedure, i.e., the delayed implant loading conditions, with 3 to 6 months of healing before prosthetic rehabilitation. A prosthetic survival rate of 98% was found with the concept proposed (3-implant system) [19]. Some authors reported more failures approaching 3 implants to support full-arch implant-fixed prosthetic restoration than using 4 or 6-implant systems [21].

The major disadvantage of fixed mandibular 3-implant retained prostheses is when one implant fails, the prosthesis is lost [20]. Bhering et al. found decreased stress levels using this system on the bone, dental implants, screws, and abutments. The magnitude of displacement of frameworks was also significantly decreased, and the authors concluded the most favorable biomechanical results were reached with stiffer materials [29].

Many studies have shown a better distribution of forces using rigid materials with a high elastic modulus and lower mass density [30]. Sirandoni et al. conducted a three-dimensional finite element analysis to investigate the biomechanical behavior of different framework materials for implant-supported fixed mandibular prostheses. According to the framework materials, the simulations were divided into six groups: titanium (Ti); cobalt-chrome (Co-Cr); zirconia (ZrO_2_); polyether ether ketone (PEEK); carbon fiber-reinforced polyether ether ketone (CFR-PEEK); and polymethyl methacrylate (PMMA). Biomechanically, PEEK and PMMA had fewer rigid models, resulting in the highest values for deformation. Conversely, stresses observed in the cortical bone were within physiologic limits in the Ti, ZrO_2_, and Co-Cr frameworks. The authors concluded that the Ti, Co-Cr, and ZrO_2_ frameworks demonstrated the most promising results considering biomechanic behaviors [31].

Another three-dimensional finite element analysis was conducted by Lee et al. to compare polyether ketone–ketone (PEKK) with different framework materials for implant-supported prostheses. The geometric models were generated using FEA software, and three materials (zirconia, titanium, and PEKK) were used for simulation. The authors concluded that the framework with the low elastic modulus (PEKK) decreased the stress within the framework; however, it transferred more stress to the supra-structures of the prostheses. Observations in the present study suggest that a resilient implant-supported framework has limited shock-absorbing properties in some areas, and rigid material showed a favorable stress distribution and overall safety for prosthesis components [32]. The FEA study conducted by Tribst et al. also demonstrated that an increase in the elastic modulus of the framework reduced the stress transmitted to the implants and surrounding bone [33].

Graphene nanoparticles (GNPs) exhibit higher Young’s modulus, tensile strength, and low mass density than Ti alloy. Thus, lower stresses are transferred from the framework, increasing its resistance to failure [34]. The present study aimed to compare the stress distribution in the All-on-6 and All- on-3 implant systems supported by titanium and graphene frameworks with different implant settings and loading simulations. Various studies have proven that minor peri-implant stresses are seen with the distal implants placed within the angulation between 15 and 45 degrees [3,35,36,37,38]. Our study considered 30-degree angulation for the most distal implants as the optimal angulation producing the lowest peri-implant stress.

In the 6-implant models, the implants were placed vertically. Therefore, our study considered the application of two loading forces, a vertical and another in the oblique direction. Studies have reported that oblique load creates stresses in the buccolingual direction and produces bending movements around the implants’ framework. It could have been due to the thinner buccal and lingual bone dimensions [39,40]. In the present study, bending movements of the framework were not noticed, and peak stresses were concentrated around the neck of the most distal implants in the cortical bone, mainly in the oblique loading.

Additionally, the stresses were seen around the trabecular bone in the vertical loading, independently of the type of framework used. This explains a higher stress value in oblique loads compared to the vertical pattern [41,42]. Similar results are seen by some authors (Sevimay et al. [43]; Jeong et al. [44]). Elsayyad et al. in their FEA study using 3-implant-supported and 4-implant-supported mandibular screw-retained prostheses, showed, under 300-N and 160-N oblique loading, that the highest stresses were recorded on the loading side at the neck of the most distal implant in the 3-implant-supported model [45], Similar stresses were also observed in our study.

In addition, Fazi et al. tested six different settings of intraforaminal implants, with the number of implants ranging from three to five, with the distal implants inserted parallelly or tilted distally (17 or 34 degrees). A prosthetic structure with 20 mm posterior cantilevers was designed for the parallel implant configurations, and a load of 200 N was applied to its distal section. In the cortical and cancellous bone, von Mises stresses resulted in 94 MPa and 125 MPa, respectively [38]. The graphene material was not tested for its ability to function as a structural material. Otherwise, we found 33.92 MPa and 1.84 MPa for the cortical and cancellous bone stresses around three implants with a titanium framework. This stress reduction can be attributed to tilting the distal implants at 30 degrees. As a result of using graphene as a support structure, we observed a 17% reduction in cortical stresses compared to titanium.

Our study used cantilevers that were 10 mm in length. In van Zyl et al.’s study, the authors found that an optimal cantilever could extend up to 15 mm. Beyond this limit, the von Mises stresses may be considered high, especially in the buccolingual region, and would be deleterious for the marginal bone level and osseointegration of the implant [46]. As the cantilever length used in our study was within the normal range, the generation of von Mises stresses was comparably low, and the distribution of the stresses to the underlying bone was safest.

Considering the framework material used, our results showed a significant decrease in the cortical stresses of 17.30% under oblique loading, which was seen for the graphene framework compared with the titanium framework. Then, graphene as a framework material showed better stress-bearing capacity when compared with the titanium framework.

We assumed that the implants were 100% osseointegrated, considering delayed implant loading conditions. To simplify the analysis, all biomaterials were supposed to be homogeneous, isotropic, and linearly elastic. None were observed in the clinical scenario. Nevertheless, there are inherent limitations to the FEA study because it is governed by boundary conditions, type of osseointegration, loading patterns, and the amount of force applied. We were unable to conduct a convergence study. Convergence analysis cannot be used to analyze three-dimensional problems. This can only be applied to two-dimensional studies. Three-dimensional studies require large amounts of computation time and heavy amounts of memory, making convergence studies impossible. The optimum size of the mesh is considered an accepted standard in finite element problems. Additionally, mesh sensitivity was not carried out. It is impossible to conduct a sensitivity study for a complicated three-dimensional problem. Sensitivity is suitable where the number of elements is lesser.

In this study, there was no experimentation, as dental studies require sensitive equipment due to the smaller load range. However, usually, all the experimental equipment is available for large load applications, and this study requires a lower load than 100 kg, for which experimentation is difficult to be conducted. In the present problem, theoretical validation was not done because it was not the perspective of the study.

Moreover, the success of the implant is largely determined by micromotion at the bone-implant interface rather than the loading timing. Furthermore, our study did not consider micromotion at the interface between the bone and the implant. Our study did not take into account a variety of variables. These variables included parafunctional behavior, oral biofilms, pH variations, fatigue effects, temperature variations, a vertical mismatch between the superstructure and abutments, and Tresca stress [47]. This study did not conduct any in vitro experiments to validate the numerical model. The current stress analysis may need to be supplemented with additional studies using photoelasticity, strain gauges, or computer imaging analysis to confirm or deny the differences observed [48,49]. Although FEA has some limitations, it offers several advantages over in vivo research methods due to the researcher’s ability to manually alter the model geometry, loading conditions, and boundary conditions on a computer. Furthermore, this method offers a significant advantage over in vivo techniques due to its repeatability [50]. Even though the results of this FEA provide an extensive understanding of the stress distribution, it is imperative to evaluate carefully before extrapolating them to further dental research or application.

## 5. Conclusions

The current study compared the outcome of von Mises stresses generated on All-on-6 and All-on-3 implant models and their respective framework biomaterials. Based on the results of the study, within its limitations, the following conclusions can be drawn:

(i) Peri-implant stresses were decreased in the graphene framework models when compared to titanium models;
(ii) Significant decrease in the cortical stresses was seen in the graphene models compared to titanium under oblique load;
(iii) The graphene framework’s load-bearing capacity was greater than the titanium framework.

Further research may be developed, including dynamic loads and nonlinear contact conditions, where nonlinear solvers can be used.

## Figures and Tables

**Table 1:** Mechanical properties of Titanium, Graphene, and Bone used for FEA [18,22].

	Thermal Conductivity (W/Mk)	Elastic Modulus (GPa)	Tensile Strength (GPa)	Poisson’s Ratio	Mass Density (kg/m^3^)	Shear Modulus (GPa)
Titanium	20	102	240	0.32	4420	45
Graphene	3000	1000	130	0.19	2270	53
Cortical bone	-	14	-	0.30	-	-
Trabecular bone	-	1.47	-	0.30	-	-

## Data Availability

Not applicable.
